# Long-term sensorimotor adaptation in the ocular following system of primates

**DOI:** 10.1371/journal.pone.0189030

**Published:** 2017-12-04

**Authors:** Markus A. Hietanen, Nicholas S. C. Price, Shaun L. Cloherty, Kostas Hadjidimitrakis, Michael R. Ibbotson

**Affiliations:** 1 National Vision Research Institute, Australian College of Optometry, Carlton, Victoria, Australia; 2 ARC Centre of Excellence for Integrative Brain Function, University of Melbourne, Parkville, Victoria, Australia; 3 Department of Optometry and Vision Sciences, University of Melbourne, Parkville, Victoria, Australia; 4 Department of Physiology, Monash University, Victoria, Australia; Tokai University, JAPAN

## Abstract

The sudden movement of a wide-field image leads to a reflexive eye tracking response referred to as short-latency ocular following. If the image motion occurs soon after a saccade the initial speed of the ocular following is enhanced, a phenomenon known as post-saccadic enhancement. We show in macaque monkeys that repeated exposure to the same stimulus regime over a period of months leads to progressive increases in the initial speeds of ocular following. The improvement in tracking speed occurs for ocular following with and without a prior saccade. As a result of the improvement in ocular following speeds, the influence of post-saccadic enhancement wanes with increasing levels of training. The improvement in ocular following speed following repeated exposure to the same oculomotor task represents a novel form of sensori-motor learning in the context of a reflexive movement.

## Introduction

When sudden, unexpected displacements of a large, textured image are presented to primates, reflexive, stereotypical ocular following responses occur, which begin the process of stabilizing the image of the moving scene on the retina (monkeys[1]; humans [2]). There are two features of these responses of particular note here. First, the initial speeds of the tracking eye movements are enhanced if the image motion occurs immediately after a saccade: post-saccadic enhancement [3]. This is interesting because it reveals that a reflexive oculomotor action is influenced by a preceding motor event. While the visual motion stimulus generated by the saccade sweeping the retina across the scene is responsible for some of the enhancement [3], evidence suggests that there is also considerable influence from the corollary discharge (i.e., efferent copy) of the signals associated with the generation of the saccade [4–6]. Corollary discharges are internal facsimiles of motor commands, which provide information to the brain about motor actions [7–10].

Second, an experiment was conducted in which monkeys were exposed for 3 days to repeated double speed steps that generated ocular following [11]. The stimuli were designed to induce visual errors by consistently varying the speed or direction of the second step from that of the first. After 3 days of exposure to the double steps, post-saccadic ocular following responses were tested with single speed steps. It was found that the ocular following responses to the speed steps were modified in predictable ways. For example, when the second speed step was faster than the first, the speed of ocular following responses to single speed steps was consistently increased. It remains unclear over what timescales this sensori-motor learning takes place, and what behavioral advantage is provided by the post-saccadic enhancement.

Previous studies have demonstrated changes in oculomotor reflexes on timescales of a few days. We wondered how these reflexes might change as a result of training or exposure over longer timescales. We established that in the absence of a prior saccade, initial ocular following speeds increased over 20–30 repeated sessions, conducted over 60–150 days. As a result of these increases the influence of post-saccadic enhancement was reduced, presumably because eye speeds were already enhanced by the learning effect and a saturation level was attained. The learning effect occurs over a much slower timescale than previously reported oculomotor changes such as saccadic adaptation [12–14], suggesting that the visual errors are learned and incorporated into the motor plan on a range of timescales. Here, we reveal a capacity for one of the eye movement control systems in primates to be modified over long periods of repeated exposure to an oculomotor task. Having established that this learning effect occurs for short-latency ocular following, the paradigm may prove useful as a tool for investigating sensori-motor learning.

## Methods

Data were collected from two male pigtail macaques (*Macaca nemestrina*). Surgical and experimental procedures were performed in compliance with National Institutes of Health (USA) and National Health and Medical Research Council (Australia) guidelines and protocols approved by the Institutional Animal Care and Use Committee at Monash University (SOBSA/P/2008/75). Each animal was implanted with a custom titanium headpost under general anaesthesia [15]. The headpost restricted head movements and allowed precise measurements of eye position during daily experimental sessions, which lasted approximately 60 minutes.

### Visual stimuli and task

Monkeys were comfortably seated with the head stabilized and received a fruit juice reward every 0.5–1 s for maintaining fixation. Visual stimuli were presented on a Sony CPD-G520 CRT monitor spanning 40 × 30 cm (W × H) placed 57 cm in front of the monkeys. The screen had a resolution of 1024 x 768 pixels (W × H) and a frame rate of 100 Hz. Monkeys were initially trained to maintain fixation on a single red spot (the fixation target) and to make saccades to any new fixation target that appeared on the screen (only one target was present at any time). During the training phase they were not exposed to the sudden image motion that occurred in the short- or long-delay conditions described below. To obtain regular fruit juice rewards, the monkeys simply had to maintain fixation and make saccades when necessary.

**Fig 1:**
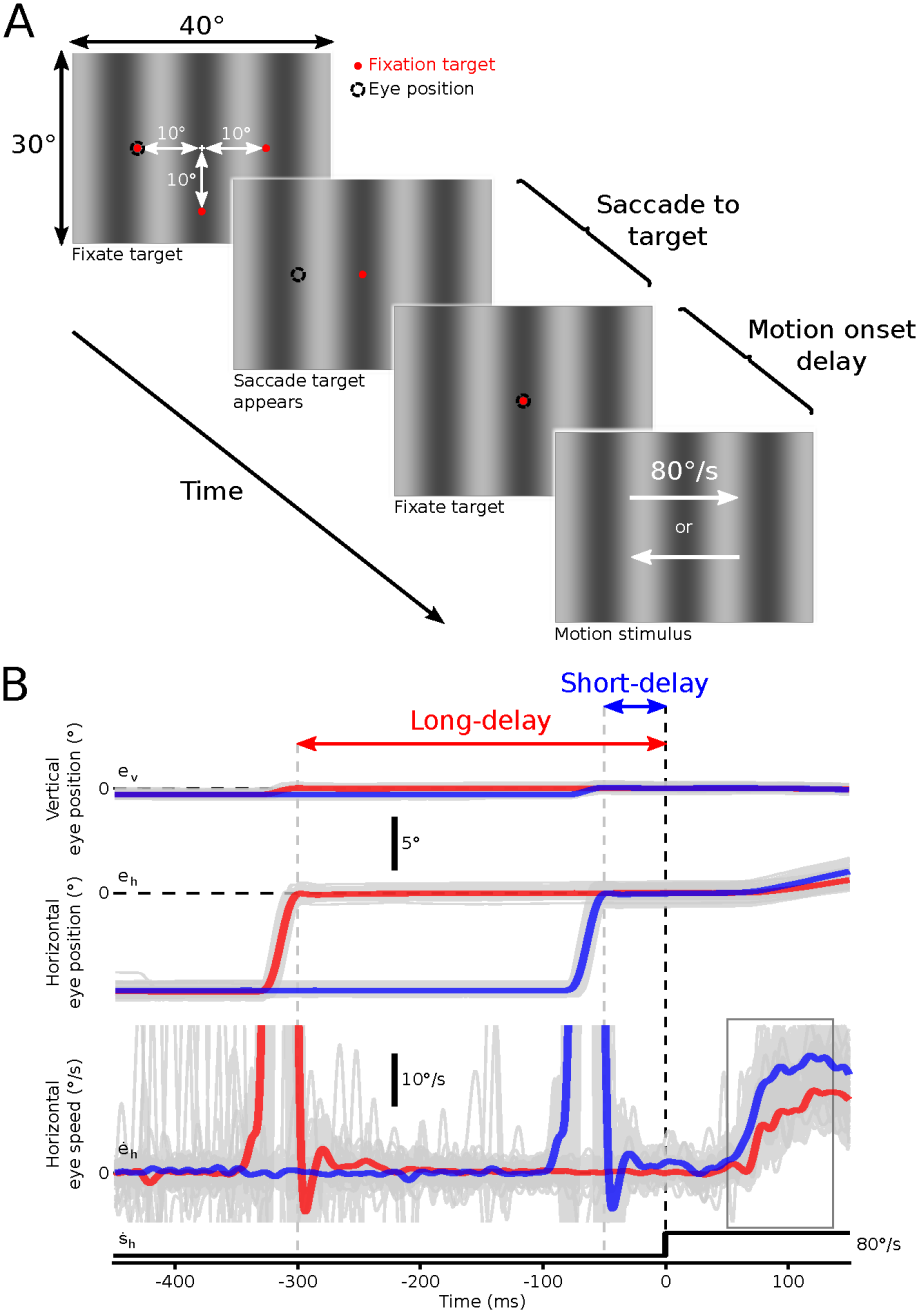
Visual stimuli, task and sample eye traces. **A**. Visual stimuli and task. Monkeys viewed vertical cosine gratings and were required to fixate a small target (red). The fixation target was initially presented 10° either to the left, to the right or below the center of the screen. This peripheral target was then removed and replaced with a central target that the monkeys were required to saccade to and fixate for either 50 ms (short-delay condition) or 300 ms (long-delay condition). At the end of this delay the grating began moving either to the left or to the right. This motion elicited robust ocular following eye movements. **B**. Sample eye traces. Example vertical (top) and horizontal (middle) eye position, and horizontal eye speed (bottom) traces from one monkey for both the short- and long-delay conditions. Eye traces for all trials (gray) were aligned at the start of the motion. Red and blue traces show mean eye position and speed signals for the long- and short-delay conditions respectively. The rectangular box on the horizontal eye speed trace indicates the analysis window for calculation of initial ocular following eye speeds.

During the experimental phase monkeys fixated one of three peripheral targets presented over a vertically oriented grating pattern with a spatial frequency of 0.781 cpd (Fig 1A). The peripheral target was either 10° to the left, right or below the center of the screen. During a trial, the peripheral target was removed and replaced with a target presented at the center of the screen. The monkey was required to make a centering saccade to this target. After the centering saccade, the monkey was required to maintain fixation for a period of either 50 ms (short-delay condition) or 300 ms (long-delay condition) after which the central fixation target was removed and the background grating moved randomly to the left or right at 80°/s for 250 ms, triggering reflexive horizontal ocular following eye movements (Fig 1B). The short-delay of 50ms was selected in order to facilitate the effect of the preceding saccade on the subsequent ocular following response, while still allowing time for the completion of the saccade prior to background motion beginning. The long delay of 300ms was selected to balance the need to minimise the effects of the prior saccade and the increasing likelihood for the monkey to break fixation as post saccadic delay increased. The monkeys were given a juice reward if they completed this sequence without making inappropriate saccades at any time. We assessed how the initial eye speed during short-latency ocular following responses changed during repeated exposure over months of testing with the same paradigm. Three parameters were varied within each daily session. First, saccade direction was randomly varied on each trial, with animals making upward, leftward or rightward saccades prior to image motion. Second, motion direction was randomly varied on each trial, with leftward or rightward image motion presented after the saccade. Third, to test for the influence of post-saccadic enhancement, we varied the motion onset delay, with the image motion starting either 50 ms (short-delay) or 300 ms (long-delay) after saccade end. These manipulations ensured that animals were unable to predict the direction or timing of the image motion and could not make systematic compensatory eye movements. Saccade direction, delay condition and motion direction were presented in random order.

All trial sequences were analysed manually following each session. Trials were excluded if there was a second saccadic eye movement between the delay trigger and the onset of motion (defined as any eye velocity exceeding 20°/s). Given the increased probability of second saccades occurring in the longer fixation period during the long-delay condition it was necessary to incorporate the presentation of twice as many long-delay than short-delay conditions to obtain approximately equal saccade counts across conditions.

### Data collection

Eye position was measured using a 1000 Hz video-based eye tracker (EyeLink 1000; SR Research, Mississauga, ON, Canada). We have previously demonstrated that the spatial and temporal precision of this eye tracker is suitable for characterizing single trial measures of ocular following [16]. For timing of the onset delay of the post-saccadic motion stimulus, saccade end was defined as that point when the eye position came within 1° of the saccade target.

### Data analysis

Eye velocity was calculated off-line using a low-pass digital differentiating filter (N = 32 ms, low-pass cut-off 80 Hz). Onset of the post-saccadic test stimulus was determined by way of a frame synchronous event marker generated by the stimulus computer. For each trial we calculated the mean eye speed in an 85 ms window beginning 50 ms after the motion stimulus onset. The start of this window corresponded to the onset of the earliest ocular following responses, defined as the earliest time after motion onset at which the eye acceleration in the direction of the motion exceeded 100°/s^2^. Enhancement was defined by taking the ratio of the short-delay condition and the long-delay condition:

$$\text{Enhancement}=\frac{eye\;speed\;\left(short\;delay\right)}{eye\;speed\;\left(long\;delay\right)}$$ (1)

## Results

The effect of training was examined by averaging initial eye speeds across all valid trials within each experimental session (one per day for each monkey) spread over a period of several weeks to months. A total of 9027 valid saccade sequences were collected from Monkey 1, and 3348 valid saccade sequences from Monkey 2. Sessions typically lasted between 30 and 60 minutes depending on the motivation and accuracy of performance of the monkey.

As the two monkeys showed different levels of motivation, Monkey 1 was tested over 154 days (21 sessions). 337 days later an additional 7 sessions (22–29) were collected between day 482 and 490, while Monkey 2 was tested over 60 days (21 sessions). Matching sessions (22–29) from Monkey 2 were not able to be obtained.

In total, four variables in our experiment could affect ocular following speed: (1) saccade direction; (2) motion direction; (3) motion onset delay; and (4) test session. Preliminary analysis of the data using a 4-way ANOVA (saccade direction, motion direction, motion onset delay and session) showed that the least significant predictor of ocular following speed was the main effect of saccade direction (monkey 1: F_2,9020_ = 13.53, p<1.36x10^-6^, μ^2^ = 0.002, monkey 2: F_2,3342_ = 26.60, p<3.46x10^-12^, μ^2^ = 0.0066). Due to the low power (μ^2^<0.01) of saccade direction as a predictor of ocular following speed, this parameter was not considered in the rest of the analysis. Instead a thorough analysis of the significant effects of saccade direction are presented as S1 Appendix, in the supplementary materials and a brief summary of these effects is presented after the main analysis.

After removing saccade direction as a predictor: A 3-way ANOVA (excluding saccade direction) was conducted for each animal.

### Monkey 1

**Fig 2:**
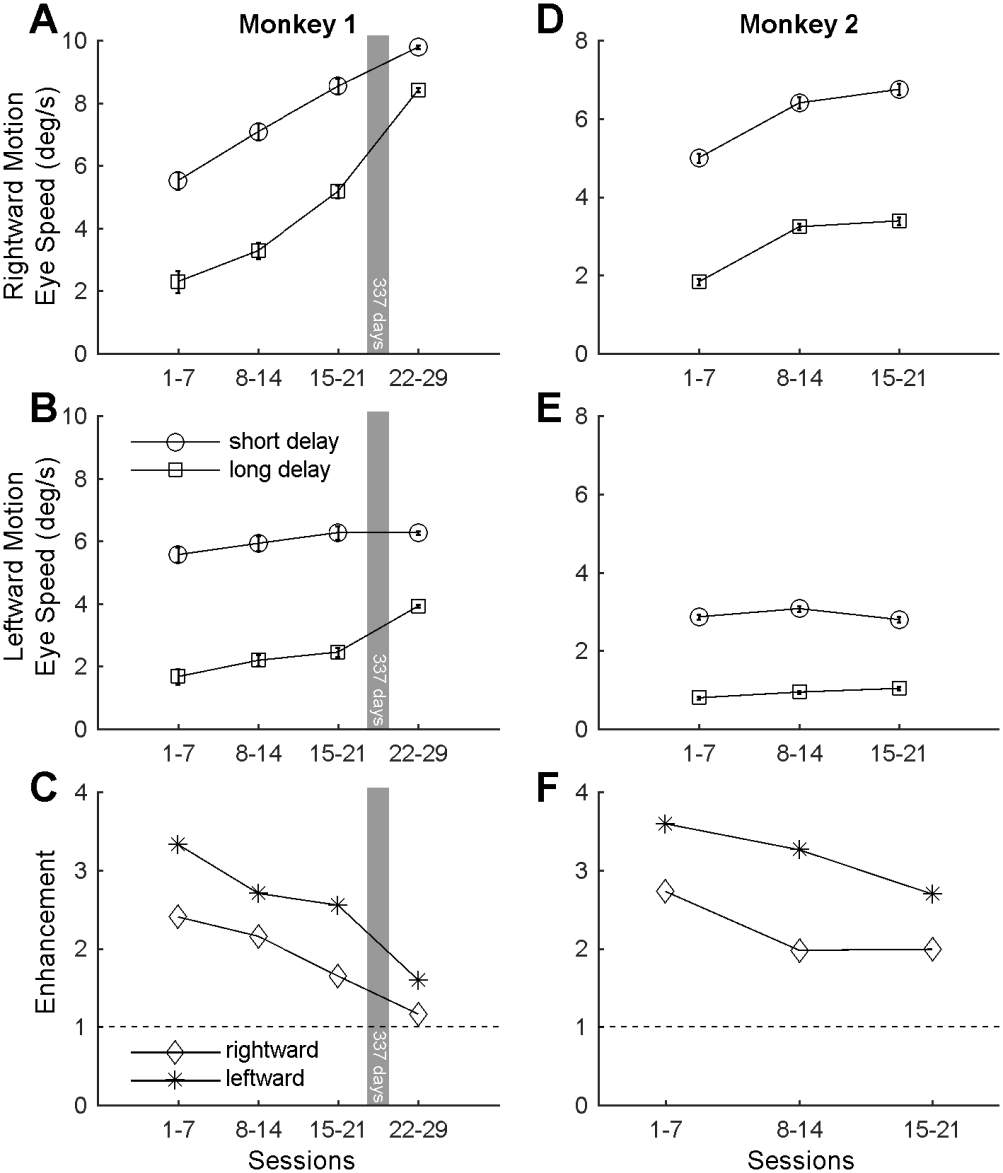
Initial eye speeds of the two monkeys over sessions. **A**,**B**. Mean ocular following eye speed for monkey 1 for rightward (**A**) and leftward (**B**) image motion plotted against binned sessions following saccades to the center of the screen at short (circles) and long (squares) delays. Error bars represent 95% confidence intervals. **D**,**E**. The same plots for monkey 2. **C**,**F**. The ratio of ocular following speed for the short-delay versus the long-delay conditions (Enhancement, Eq 1) for rightward and leftward motion in monkey 1 (**C**) and monkey 2 (**F**). In general, ocular following eye speed was faster in the short-delay condition. Over time, initial ocular following eye speed increased such that this post-saccadic enhancement of the ocular following response was completely (monkey 1) or partly (monkey 2) abolished.

A 3-way ANOVA incorporating onset delay, motion direction and session was conducted. A significant main effect of onset delay (F_1,9012_ = 1627.6, p<0.001, μ^2^<0.098) was demonstrated such that ocular following speeds for each eye and session were always faster for short delays where the saccade is more likely to have an effect on the subsequent ocular following response (initial eye speed = 7.85°/s, SEM = 0.03) than long delays where the ocular following speed is unlikely to be affected by the preceding saccade (initial eye speed = 5.73°/s, SEM = 0.04°/s). Other main effects for motion direction (F_1,9012_ = 4955.8, p<0.001, μ^2^<0.298) and session (F_1,9012_ = 336.41, p<0.001, μ^2^<0.061) were also identified. A significant 2-way interaction between motion direction and session number was found (F_1,9012_ = 108.00, p<0.001, μ^2^<0.018), so the effects of onset delay were considered separately for rightward (Fig 2A) and leftward motion (Fig 2B). A 2-way ANOVA of the effect of onset delay and session number for rightward stimulus motion showed a significant interaction (F_3,4534_ = 27.89, p<0.001, μ^2^<0.0136). Consequently, 1-way ANOVAs examining the influence of session were conducted for each onset delay. For the short-delay and rightward stimulus motion there was a significant increase in ocular following speed ($\bar{x}$°/s±SEM = 5.5±0.28, 7.1±0.27, 8.5±0.25 & 9.8±0.05) over successive binned sessions (Fig 2A, circles; F_3,2292_ = 108.85, p<0.001, μ^2^<0.125). Bonferroni-corrected pairwise comparisons showed significant differences of 1.6°/s, 1.4°/s, and 1.3°/s respectively, between successive bins of sessions (t-tests, p<0.001), demonstrating that between every set of binned sessions there was an increase in ocular following speeds immediately following a saccade. The total increase of 4.3°/s between the first sessions and the last sessions was also significantly different (t_2091_ = 15.48, p<0.001). For the long-delay condition there was a similar but stronger increase in ocular following speeds ($\bar{x}$°/s±SEM = 2.3±0.35, 3.3±0.28, 5.2±0.20 & 8.4±0.06) with session number (Fig 2A, squares; F_3,2242_ = 266.46, p<0.001, μ^2^<0.263). Bonferroni-corrected pairwise comparisons showed significant increases of 1.0°/s, 1.9°/s, and 3.2°/s respectively, over successive sessions (t-tests, p<0.001). The total increase of 6.1°/s between the first sessions and the last sessions was also significantly different (t_1962_ = 17.11, p<0.001), demonstrating a significant increase in ocular following eye speeds with time that is unlikely to have been affected by the preceding saccade.

A 2-way ANOVA of the effect of onset delay and session for leftward stimulus motion showed a significant but powerless interaction (F_3,4478_ = 21.21, p<0.001, μ^2^<0.01). There was a main effect of onset delay, revealing a significant increase in ocular following speeds following the short-delay condition ($\bar{x}$ = 6.2°/s) compared to the long-delay condition ($\bar{x}$ = 3.7°/s, F_1,4478_ = 668.84, p<0.001, μ^2^ = 0.105). There was also a significant but weaker main effect of session number (F_3,4478_ = 44.14, p<0.001, μ^2^ = 0.021). Bonferroni-corrected pairwise comparisons of binned sessions as they progressed found significant increases between the marginal means of only the last two bins (Fig 2B, 15–21 & 22–29) in the progression (t_4135_ = 7.49, p<0.0001), collected 337 days apart.

It is established that ocular following speeds are higher when image motion commences immediately after the end of a saccade.

The level of post-saccadic enhancement (Eq 1) decreases over sessions, as is evident in the ratio of ocular following eye speeds for the short- and long-delay conditions (Fig 2C). For both left- and rightward image motion the relative impact of the post-saccadic enhancement in the short-delay condition decreased over time.

### Monkey 2

Similar trends were found for the two monkeys; as for Monkey 1, a 3-way ANOVA incorporating onset delay, motion direction and session was conducted. Again the most significant main effect was that of onset delay (F_1,3337_ = 5451.9, p<0.001, μ^2^<0.305) demonstrating that ocular following speeds were significantly faster for short delays (initial eye speed = 6.05°/s, SEM = 0.14°/s) than long delays (initial eye speed = 2.82°/s, SEM = 0.09°/s). Significant main effects of motion direction (F_1,3337_ = 2053.0, p<0.001, μ^2^<0.257) and session number (F_1,3337_ = 117.57, p<0.001, μ^2^<0.030) were also found. A significant 2-way interaction between motion direction and session number was identified (F_1,3337_ = 95.63, p<0.001, μ^2^<0.022), and between onset delay and motion direction (F_1,3337_ = 148.26, p<0.001, μ^2^<0.017), so the effects of onset delay were considered separately for rightward (Fig 2D) and leftward motion (Fig 2E). A 2-way ANOVA of the effect of onset delay and session number for rightward stimulus motion showed significant main effects for both parameters (onset delay: F_1,1639_ = 1233.5, p<0.001, μ^2^<0.384; session: F_2,1639_ = 141.32, p<0.001, μ^2^<0.088). Bonferroni-corrected pairwise comparisons of binned sessions as they progressed found a significant increase of 1.41°/s between the marginal means of the bin containing the first seven sessions (initial eye speed = 3.41°/s, SEM = 0.10°/s) and the bin containing the subsequent seven sessions (initial eye speed = 4.82°/s, SEM = 0.11°/s) (t-tests, p<0.0001). There were no further significant increases in ocular following speed between the second and third bin (t-test, p>0.05).

A 2-way ANOVA of the effect of motion onset delay and session number for leftward stimulus motion showed only a significant increase (F_1,1698_ = 1617.5, p<0.001, μ^2^<0.492) for ocular following motion in the short-delay condition (initial eye speed = 2.91°/s, SEM = 0.07°/s) compared to the long-delay condition (initial eye speed = 0.93°/s, SEM = 0.04°/s). Taking the ratio of initial ocular following eye speeds for the short- and long-delay conditions (Eq 1) revealed a reduction in the relative impact of the post-saccadic enhancement (Fig 2F) as sessions progressed, as was the case for Monkey 1. Due to other experimental demands, we were unable to test Monkey 2 beyond the initial 21 sessions.

### Ocular following changes depend on the number of training sessions

**Fig 3:**
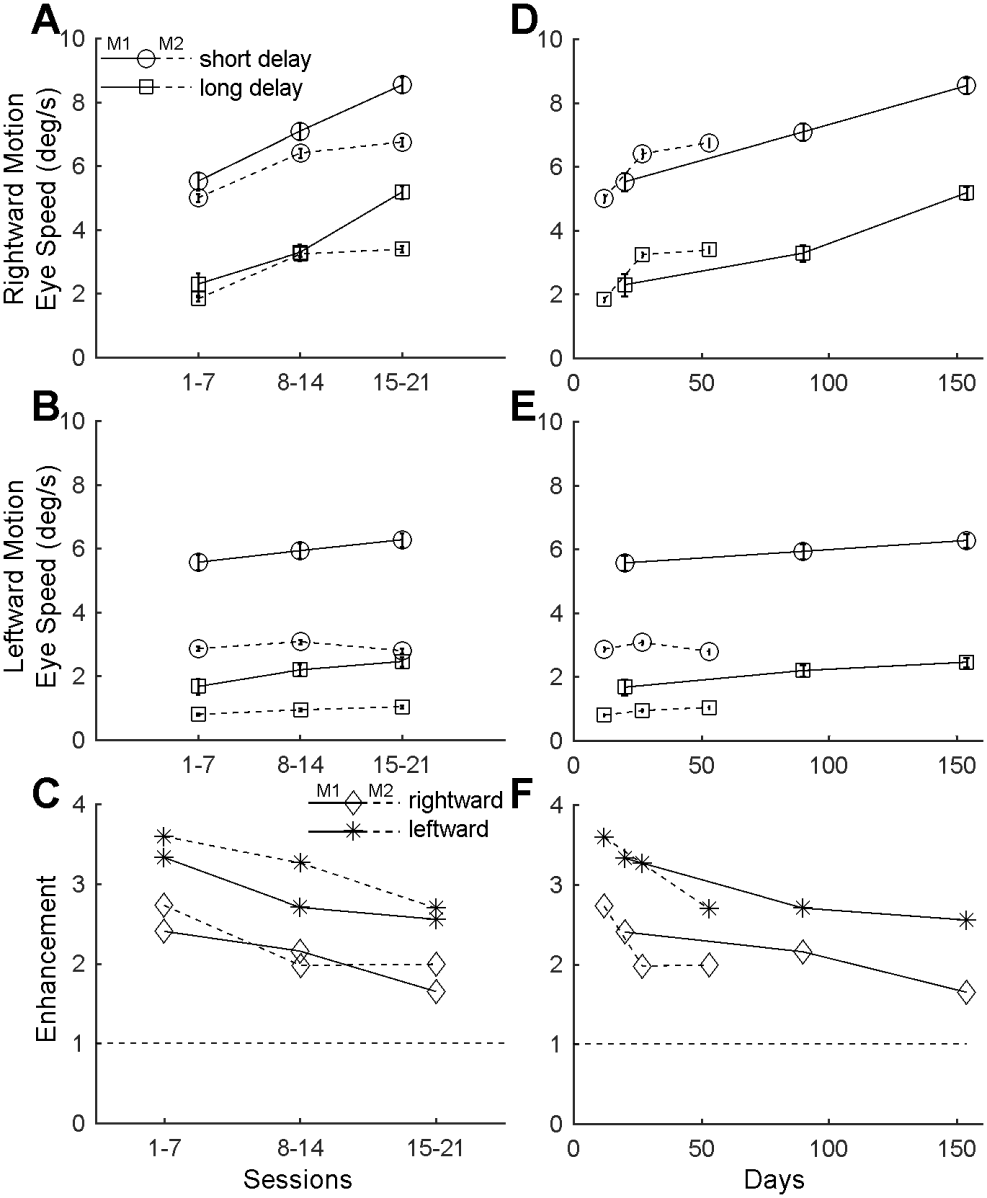
Time course of ocular following adaptation in the two monkeys. Comparison of the ocular following speed and of the post-saccadic enhancement across an equal number of sessions (A-C) and across time (D-F) between the two monkeys. Ocular following speeds increase over time (with ongoing exposure to the same paradigm), but mainly for rightward motion. Both Monkeys had slight differences in eye speed related to saccade direction, with Monkey 1 showing larger and sustained increases over time in ocular following responses for rightward background motion, and Monkey 2 reaching peak ocular following responses for rightward motion over fewer sessions.

So far, we have assessed the change in initial ocular following speed in relation to session number. This revealed reasonable consistency between the two monkeys. However, as the animals had different inherent levels of motivation, i.e. M1 was more reluctant to perform the task than M2, the collection of comparable amounts of data required larger inter-session times. As such, Monkey 1 was tested over a longer period (154 days; 21 sessions) than Monkey 2 (60 days; 21 sessions). Replotting the data against session number (Fig 3A–3C) and directly comparing it with the data plotted against days (Fig 3D–3F) suggests that the training effect occurred more rapidly when the sessions were closer together (Monkey 2) but reached similar levels of enhancement after similar numbers of sessions (21 sessions). The data obtained from M1 suggests that this training effect seems to persist even following a gap between sessions 21 and 22 of over 7 months. Unfortunately, M2 was no longer available for testing after the 21st session to allow for replication between animals.

### Ocular following eye speed changes involving saccade direction

To determine whether visual stimulation during a saccade would influence the ocular following response, we presented stationary vertical gratings during both horizontal and vertical saccades. As the gratings were vertical and stationary during saccades, upward saccades should not have presented a large visual drive prior to the onset of background motion as the saccade is along the grating. In contrast leftward and rightward saccades could hypothetically provide an apparent motion stimulus during the saccade that may affect the subsequent ocular following response speeds. During a rightward saccade across a vertical grating the apparent motion on the retina would be leftward and vice-versa. A rightward saccade, would lead to leftward apparent motion. We would then expect reduced ocular following speeds for leftward background motion and/or increased ocular following speeds for rightward motion. The opposite effect should hypothetically be present for leftward saccades. The vertical saccades serve as a useful baseline to compare the interaction between leftward and rightward saccades and 3 possible outcomes are possible:

1. Post saccadic enhancement is unaffected by the interaction between the apparent motion due to a saccade and the motion of the background after the saccade;
2. Post-saccadic enhancement improves tracking of the background over baseline. In this case tracking in the same direction as the saccade should be faster than both tracking in the opposite direction to the saccade and to the upward saccades;
3. Post-saccadic enhancement offsets a deficit in eye-speed induced by the apparent motion during the saccade. In this case tracking in the opposite direction to the saccade would be slower than both tracking in the same direction as the prior saccade and tracking following an upward saccade.

**Fig 4:**
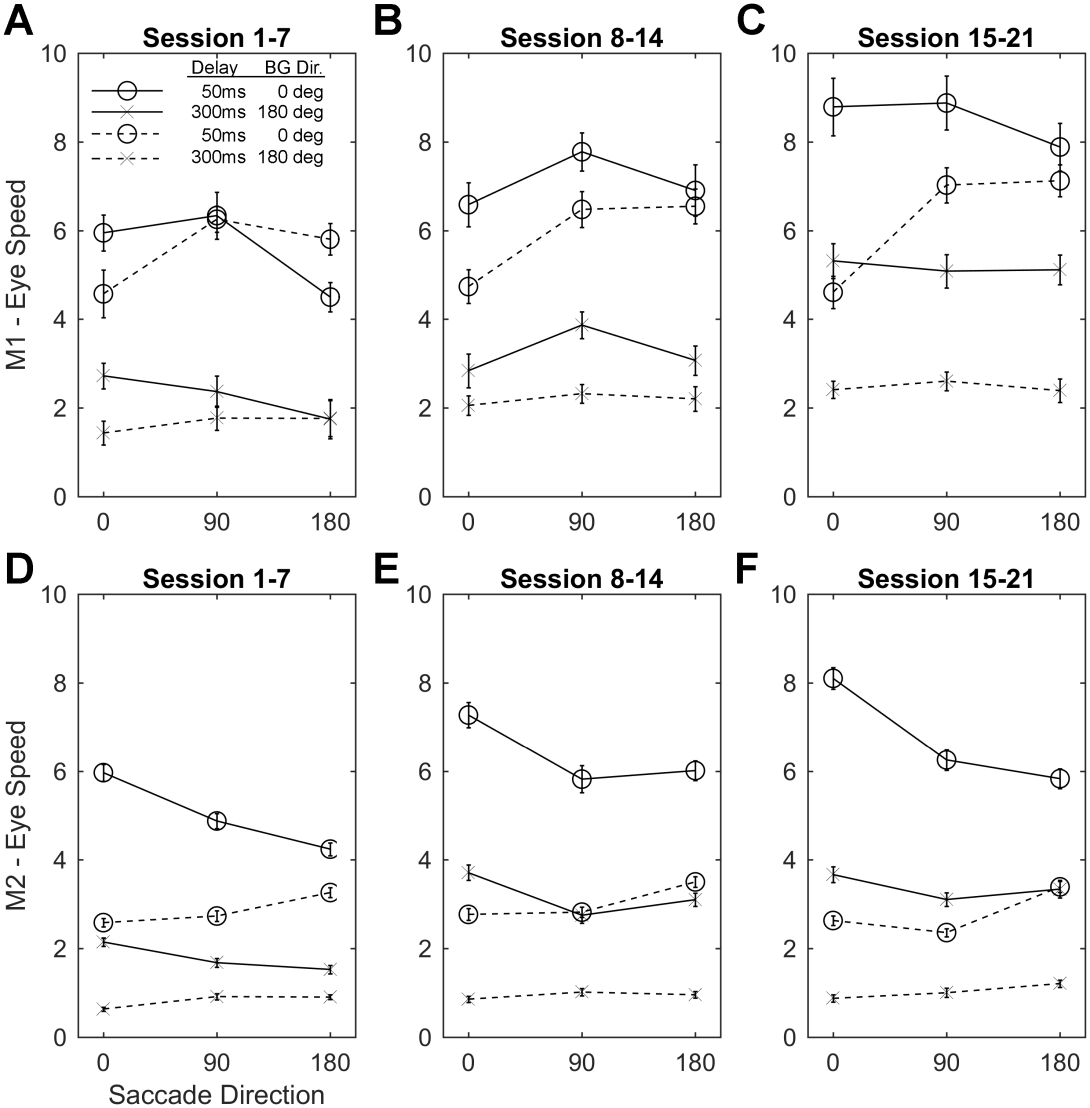
Analysis of ocular following speed following saccades at 0, 90 and 180 degrees. Comparison of the ocular following speed for Monkey 1 (A-C) and Monkey 2 (D-F). Each plot: 1) shows the saccade direction across a stationary vertical grating (abscissa) plotted against the ocular following eye speed (ordinate); 2) Open circles show the responses at short delay (50ms) conditions, while the x’s show the responses at the long delay (300ms) conditions; 3) Solid and dashed lines indicate that the background motion driving the ocular following response was rightward (0deg) or leftward (180deg) respectively. Monkey 1 shows a tendency for ocular following responses to be reduced when the background motion during ocular following is in the opposite direction to the preceding saccade, compared to the same or vertical conditions. This effect is strongest and equal for short delay conditions (circles) in the first 7 sessions (A). Monkey 2 shows a tendency for ocular following responses to increase when background motion is in the same direction as the preceding saccade, compared to the opposite and vertical directions. This effect is strongest for the short delay conditions (circles 50ms) and is consistently strong across sessions (D-F).

While our results (Fig 4 & S1 Appendix) demonstrate that there are significant interactions between saccade direction and background motion direction. For Monkey 1 the pattern of post-saccadic enhancement varied in a pattern consistent with the third outcome outlined above, particularly for the first 7 sessions where the effect of saccade direction explained 11.61% and 9.02% of the variability in eye-speed for rightward and leftward background motion respectively but only for the 50ms delay condition. This result was partially replicated in sessions 15–21 where 21.7% of the variability in ocular following speeds for rightward motion were reduced following leftward saccades. For Monkey 2 the pattern of post-saccadic enhancement varied in a pattern consistent with the second example outcome outlined above. Ocular following speeds where the direction of the saccade and background motion were the same were significantly increased in every condition, compared to when saccades were upward or in the opposite direction to the subsequent background motion. Saccade direction explained between 5.95% & 20.03% of the variability in eye speed in short post-saccadic delay conditions, consistently across time. While for the long delay conditions saccade direction explained between 0.75%(NS) and 6.09% of the variability in post-saccadic eye speeds. Importantly in both monkeys there was a small but significant differential effect of visual stimulation during the saccade on subsequent ocular following speeds.

## Discussion

We have observed two new changes in ocular following that occur over many weeks of testing. Primarily, the speed of short-latency tracking eye movements increases with training over long periods but appears to be dependent more on the total exposure (sessions) to the ocular following stimulus presented, and unaffected by the passage of time during which testing was not conducted. Unfortunately the training schemes of both of our monkeys were not similar with M1 taking 150 days, and M2 taking 60 days to complete 21 sessions. As a result, future research may be required to replicate our findings that the training effect isn’t affected by intersessional delays. Additionally, we were unable to collect data after the 21st session from M2, as we had from M1. This result should also be considered tentative until it is replicated. Furthermore, the post-saccadic enhancement of ocular following speed correspondingly diminishes. In both animals the direction of a centering saccade (left, right or up) had no effect on changing the initial speeds of post-saccadic ocular following responses. For both animals, ocular following speeds were also significantly higher for short versus long post-saccadic delays, regardless of the direction of the saccade or image motion. This provides strong support for the existence of post-saccadic enhancement in the paradigm used. Ocular following speeds were faster in both animals for rightward versus leftward image motion. Given that all recordings were obtained from each animal’s right eye, this indicates a tendency for higher ocular following speeds when the eye is tracking from nasal-to-temporal. Similar asymmetries in ocular following speeds for leftward and rightward stimuli have been noted in both monkey and human subjects previously [1–2], however as the present study did not collect data from the left eye during recordings we are unable to comment beyond the fact that the asymmetry in ocular following responses between responses to leftward and rightward background motion do not conflict with prior studies. Most importantly in the context of the present paper, both animals showed slow but significant increases in ocular following speed as time progressed when rightward motion was presented to the right eye. This finding was also found for leftward motion in Monkey 1. The increase in ocular following speed occurred both when the tracking occurred immediately after a saccade (short-delay condition) or long after a saccade (long-delay condition). The increase in initial speed was great enough that the influence of post-saccadic enhancement was reduced to quite low levels after 20–29 test sessions, suggesting that there is a physical limit on how fast the initial eye speeds can be. Our results also suggest against comparing results across time when using short-latency ocular following paradigms, as the amplitude of the baseline reflex is not constant.

What mechanism could be at work to slowly increase initial ocular following speeds over days, weeks and months of training? What makes this question particularly interesting is that the monkeys never gain any particular advantage from the reflexive ocular following. The image motion only lasts 150 ms and eye speeds are never able to catch up with the stimulus in that short time. Therefore, initial enhancement does not obviously lead to improved image stability. Despite this lack of feedback, the system improves its initial speeds.

By exposing monkeys for 3 days to repeated motion stimuli that generated ocular following and induced visual errors, Miles and Kawano showed that the animal’s ocular following responses were subject to visually mediated adaptive change [11]. This finding revealed that some type of plasticity occurs in the oculomotor reflex but the authors did not differentiate between the basic ocular following reflex and post-saccadic enhancement. In their experiments, ocular following always occurred 50 ms after a saccade, so all ocular following was subject to post-saccadic effects. Experiments in which a saccade was made to a moving target, which was then required to be tracked by the subject using smooth pursuit, have shown that the retinal slip during the smooth pursuit generates saccadic adaptation [17]. This implies that tracking responses, such as smooth pursuit and short-latency ocular following have the capacity to future influence saccades. However, in our paradigm we were able to show that a prior saccade was not necessary to induce the increases in ocular following speeds, so it is unlikely that saccadic adaptation has a role in this effect.

Even though the Havermann study [17] relates to saccadic adaptation and smooth pursuit, which use circuits that differ from those involved in short-latency ocular following [14,18], the finding that retinal slip speed had an important adaptive effect has a bearing on the present findings. Also, they point out that the signal that drove the saccadic adaptation is dissociated in time from the error signal (retinal slip) that induced the change. That is, only when the next saccade to a moving target occurred could any saccadic changes be seen. This is similar to our paradigm. The increase in initial ocular following speeds depended entirely upon the exposure to retinal slip during previous ocular following sessions, which might have occurred days or weeks earlier. Therefore, we argue that the effect we have observed represents a form of motor learning. A similar form motor learning has been demonstrated in smooth pursuit tasks occurring at a variety of time scales, including within a single trial (subsequent catch-up saccades are reduced in size), across a session, and from session-to-session [19]. They demonstrated that pursuit eye-movement behaviours are acquired, with initial trials and sessions showing a larger number of catch up saccades. Our study demonstrates that initially the ocular motor system shows enhancement immediately following a saccade (in our ocular following task) and that this enhancement diminishes over sessions. This post saccadic enhancement of tracking may provide an insight into why catch-up saccades are favoured in early sessions of smooth pursuit tasks such as those used by Bourelly and colleagues.

Oculomotor plasticity has previously been reported in the context of the Optokinetic Response (OKR), vestibulo-ocular reflex (VOR), smooth pursuit and saccades. Collectively, these adaptations help improve the stability of the visual scene on the retina. The OKR generates eye rotation to stabilise the visual image on the retina by turning in the direction of the motion of the visual world. The VOR generates eye rotation that opposes head movements to stabilise the visual world on the retina [20–21]. If the counter-rotating eye and head movements are not perfectly matched (e.g. because of the introduction of lenses or systematic stimulus manipulation) a visual “teaching” signal indicates that the gain and phase of the eye movements should be updated. This process of learning a new visual-vestibular relationship is evident within 30 minutes—much shorter than the timescales we report. Critically, it only saturates after many days, and is hypothesised to depend on long-term depression in the cerebellum [22–25]. Smooth pursuit adaptation is commonly demonstrated by having subjects volitionally track a moving target, which undergoes a small step change in speed 100–200 ms after the initial onset of motion [26–27]. After prolonged exposure to these step changes, eye acceleration changes, effectively anticipating the step change. As in VOR learning, smooth pursuit adaptation is evident within a few hundred trials ([27]) and is dependent on the dorsolateral pontine nucleus [28] and cerebellar vermis [29].

A third type of oculomotor plasticity is evident in saccade adaptation, which can be generated by shifting a saccade target while the eye is in motion; consistent undershoots (or overshoots) in final eye position relative to the target lead to compensatory decreases (increases) in saccade size after 1000–2000 saccades [12–13]. Complete compensation for saccadic errors can be stably achieved within a testing session lasting an hour, and depend on the oculomotor vermis and caudal fastigial nucleus in the cerebellum [14,30]. In addition to this short term saccade adaptation, a longer-term effect was reported when macaques made 1000–3000 saccades in daily testing sessions over 3 weeks, but were blindfolded during non-training periods [31]. The neuronal basis of this slower adaptation is unclear.

Intriguingly, the timescales of previously-reported adaptation effects vary markedly from hundreds of trials through to many days. A distinguishing feature of the phenomenon that we report is that it arises simply through repetition of the “normal” reflex, whereas previous studies have deliberately manipulated the sensory inputs. The adaptation that we have observed is also particularly striking because the long-term manipulation that we apply is not as severe as in other contexts. For example, in studies of long term saccade adaptation, animals were blindfolded when not being tested [31], and studies of VOR adaptation have animals wear lenses for many months [32].

The size of the effect we have observed suggests that adapting the ocular following reflex is critical for ensuring stability of the visual image. Where might this effect originate? Learning in the VOR depends on the cerebellum [21, 33, 34], and may specifically depend on a mechanism of long-term depression that involves the metabotropic glutamate receptor subtype 1 (mGluR1) [23]. Given that VOR learning depends on a similar visual error signals to our protocol, we argue that the cerebellum is one of the sites that undergo plasticity during ocular following adaptation. In line with this view, computational work by Yamamoto and colleagues [35–36] showed that simulated plastic changes in the synaptic inputs of Purkinje cells in the cerebellar paraflocculus could produce ocular following adaptation. Given that VOR learning depends on a similar visual error signal to our protocol, we argue that cerebellar long-term depression may also underpin adaptation in ocular following.

### Visual stimulation during a saccade influences subsequent ocular following responses

We hypothesised that if visual stimulation during a saccade were to influence the post-saccadic enhancement of the ocular following response [3], then it would be demonstrable by either; 1) an increased ocular following speed when tracking a background moving in the same direction as the saccade or 2) a reduced ocular following speed when tracking a background moving the opposite direction as the saccade. We used vertical saccades as a control where the visual drive during a saccade would be small as the saccade would be along the contour of the grating rather than across it. We found each of our monkeys displayed one of these patterns of effects, particularly when the delay between saccade and background motion onset was short. This demonstrates that there is a small but significant visual effect on the short latency ocular following response as was also shown by Cloherty and colleagues [6].

## Supporting information

S1 Data This file contains the values used for conducting the analyses presented in the main results. (XLSX)

S1 Appendix This file contains an explanation of the analysis and results of the data while allowing saccade direction to remain as a predictor variable. (DOCX)
