# Supplementary material for: Long-term sensorimotor adaptation in the ocular following system of primates
Source: PLoS One. 2017 Dec 4;12(12):e0189030. doi: 10.1371/journal.pone.0189030 (PMC5714349; doi:10.1371/journal.pone.0189030)
Supplement: S1 Appendix — (DOCX) [file pone.0189030.s002.docx]

# APPENDIX A: An analysis of post-saccadic ocular following speed including saccade direction as a predictor.

## A brief explanation of the presentation of the results in the following tables.

1. All text in red are non-significant results.
2. All text highlighted in yellow provide a description of the analysis decision process based on the results of the table immediately before it that determined how the analysis should proceed.
3. All text highlighted in green describe the endpoint of the analysis and the final statistical evidence for the significant effect found in prior tables.
4. The presented figures correspond to the text in green and are in the same order.
5. Eta^2^ is the proportion of the total variability in eye speed that can be accounted for by the variable being considered for example in the table below Delay has an Eta^2^ of 0.3036, which indicates that 30.36% of the differences in eye speed can be accounted for by the main effect of post-saccadic delay.

The following key is useful when looking at the figures.

- Motion = 0 is rightward (0deg) background motion following the post-saccadic delay.
- Motion = 1 is leftward (180deg) background motion following the post-saccadic delay.
- Saccade = 0 is a rightward (0deg) saccade over a stationary background.
- Saccade = 1 is a upward (90deg) saccade over a stationary background.
- Saccade = 2 is a leftward (180deg) saccade over a stationary background.
- Delay = 0 is a 50ms post-saccadic delay between saccade onset and background motion.
- Delay = 1 is a 300ms post-saccadic delay between saccade onset and background motion.

# Reminder of our hypotheses and associated experimental details

1. We are using ocular following speed as an indicator of the level of post-saccadic enhancement of the visual system.
2. We expect that ocular following speeds will be faster 50ms after a saccade than 300ms after a saccade.
3. Gratings were vertical and stationary during saccades. Therefore, upward saccades shouldn’t have a large visual drive present prior to the onset of motion. While leftward and rightward saccades could hypothetically provide an apparent motion stimulus during the saccade that would affect the subsequent ocular following response speeds.
4. During a rightward saccade across a vertical grating the apparent motion on the retina would be leftward and vice-versa.
5. A rightward saccade, would lead to leftward apparent motion. We would then expect reduced ocular following speeds for leftward background motion and/or increased ocular following speeds for rightward motion. The opposite effect should be present for leftward saccades.
6. We would expect this effect to be strongest for shorter post-saccadic delays due to enhancement of the visual system.
7. The vertical saccades serve as a useful baseline to compare the interaction between leftward and rightward saccades and 3 possible outcomes are possible:
   1. Post saccadic enhancement is unaffected by the interaction between the apparent motion due to a saccade and the motion of the background after the saccade;
   2. Post-saccadic enhancement improves tracking of the background over baseline. In this case tracking in the same direction as the saccade should be faster than both tracking in the opposite direction to the saccade and to the upward saccades;
   3. Post-saccadic enhancement offsets a deficit in eye-speed induced by the apparent motion during the saccade. In this case tracking in the opposite direction to the saccade would be slower than both tracking in the same direction as the prior saccade and tracking following an upward saccade.
8. The results as presented below demonstrate that in Monkey 1 we found that hypothesis (c) explained the data better, while in Monkey 2 hypotheses (b) was a better fit to the data.
9. In all cases eye speeds were faster for short delays (50ms) than long delays (300ms), and both animals had a bias for faster rightward eye-motion.
10. The results do match the patterns that we hypothesized, however the effects are very small compared to the effects outlined in point 9 above.

# MONKEY 1: 4-WAY ANOVA

'Source' 'Sum Sq.' 'd.f.' 'Singular?' 'Mean Sq.' 'F' 'Prob>F' 'Eta2' 'Partial Eta2'

'Delay' [3.7412e+03] [ 1] [ 0] [3.7412e+03] [679.2000] [2.6640e-119] [ 0.3036] [ 0.3586]

'Motion' [ 484.5057] [ 1] [ 0] [ 484.5057] [ 87.9605] [ 3.1583e-20] [ 0.0393] [ 0.0675]

'Saccade' [ 101.9718] [ 2] [ 0] [ 50.9859] [ 9.2563] [ 1.0241e-04] [ 0.0083] [ 0.0150]

'Time' [ 645.1667] [ 2] [ 0] [ 322.5834] [ 58.5640] [ 5.2291e-25] [ 0.0524] [ 0.0879]

'Delay*Motion' [ 6.4866] [ 1] [ 0] [ 6.4866] [ 1.1776] [ 0.2781] [5.2635e-04] [ 9.6830e-04]

'Delay*Saccade' [ 51.9784] [ 2] [ 0] [ 25.9892] [ 4.7183] [ 0.0091] [ 0.0042] [ 0.0077]

'Delay*Time' [ 2.7094] [ 2] [ 0] [ 1.3547] [ 0.2459] [ 0.7820] [2.1985e-04] [ 4.0468e-04]

'Motion*Saccade' [ 105.2689] [ 2] [ 0] [ 52.6344] [ 9.5556] [ 7.6269e-05] [ 0.0085] [ 0.0155]

'Motion*Time' [ 235.3915] [ 2] [ 0] [ 117.6957] [ 21.3673] [ 7.5818e-10] [ 0.0191] [ 0.0340]

'Saccade*Time' [ 24.3481] [ 4] [ 0] [ 6.0870] [ 1.1051] [ 0.3526] [ 0.0020] [ 0.0036]

'Delay*Motion*Sacc…' [ 50.6332] [ 2] [ 0] [ 25.3166] [ 4.5961] [ 0.0103] [ 0.0041] [ 0.0075]

'Delay*Motion*Time' [ 5.9430] [ 2] [ 0] [ 2.9715] [ 0.5395] [ 0.5832] [4.8224e-04] [ 8.8723e-04]

'Delay*Saccade*Time' [ 7.7221] [ 4] [ 0] [ 1.9305] [ 0.3505] [ 0.8438] [6.2660e-04] [ 0.0012]

'Motion*Saccade*Time' [ 26.3190] [ 4] [ 0] [ 6.5798] [ 1.1945] [ 0.3114] [ 0.0021] [ 0.0039]

'Delay*Motion*Sacc…' [ 10.2906] [ 4] [ 0] [ 2.5727] [ 0.4671] [ 0.7600] [8.3502e-04] [ 0.0015]

'Error' [6.6925e+03] [1215] [ 0] [ 5.5082] [] [] [ 0.5430] []

'Total' [1.2324e+04] [1250] [ 0] [] [] [] [] []

Given that we have a 3-way interaction that doesn’t involve Time(Sessions) so let’s have a look at what the data looks like at each group of sessions:

## MONKEY 1: 3-WAY ANOVA Sessions 1-7

'Source' 'Sum Sq.' 'd.f.' 'Singular?' 'Mean Sq.' 'F' 'Prob>F' 'Eta2'

'Delay' [ 909.4873] [ 1] [ 0] [909.4873] [227.1938] [3.7134e-38] [0.4436]

'Motion' [ 7.9883] [ 1] [ 0] [ 7.9883] [ 1.9955] [ 0.1589] [0.0070]

'Saccade' [ 26.6698] [ 2] [ 0] [ 13.3349] [ 3.3311] [ 0.0372] [0.0228]

'Delay*Motion' [ 5.7574] [ 1] [ 0] [ 5.7574] [ 1.4382] [ 0.2314] [0.0050]

'Delay*Saccade' [ 14.1776] [ 2] [ 0] [ 7.0888] [ 1.7708] [ 0.1721] [0.0123]

'Motion*Saccade' [ 46.1824] [ 2] [ 0] [ 23.0912] [ 5.7683] [ 0.0035] [0.0389]

'Delay*Motion*Sacc…' [ 5.5600] [ 2] [ 0] [ 2.7800] [ 0.6945] [ 0.5002] [0.0048]

'Error' [1.1409e+03] [ 285] [ 0] [ 4.0031] [] [] [0.5293]

'Total' [2.1553e+03] [ 296] [ 0] [] [] [] []

Since there is no interaction due to delay let’s have a look at the data at each delay

### MONKEY 1: 2-WAY ANOVA Sessions 1-7, Delay = 50ms

'Source' 'Sum Sq.' 'd.f.' 'Singular?' 'Mean Sq.' 'F' 'Prob>F' 'Eta2'

'Motion' [ 0.1167] [ 1] [ 0] [ 0.1167] [0.0231] [0.8795] [1.3171e-04]

'Saccade' [ 46.4166] [ 2] [ 0] [ 23.2083] [4.5846] [0.0115] [ 0.0498]

'Motion*Saccade' [ 56.0471] [ 2] [ 0] [ 28.0235] [5.5358] [0.0047] [ 0.0595]

'Error' [885.8914] [ 175] [ 0] [ 5.0622] [] [] [ 0.8968]


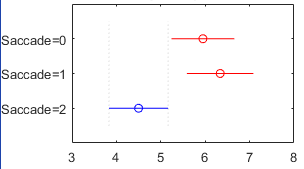
 'Total' [987.7816] [ 180] [ 0] [] [] [] []

Since there is an interaction between Motion & Saccade let’s have a look at the data for each motion direction

#### MONKEY 1: 1-WAY ANOVA Sessions 1-7m Delay = 50ms, Motion = 0

'Source' 'Sum Sq.' 'd.f.' 'Singular?' 'Mean Sq.' 'F' 'Prob>F' 'Eta2'

'Saccade' [ 57.0477] [ 2] [ 0] [ 28.5239] [5.5825] [0.0053] **[0.1161]**

'Error' [434.3093] [ 85] [ 0] [ 5.1095] [] [] [0.8839]

'Total' [491.3571] [ 87] [ 0] [] [] [] []

There is a significant reduction in eye speed between Saccades at 180deg and Saccades at 0 or 90degs.

#### MONKEY 1: 1-WAY ANOVA Sessions 1-7m Delay = 50ms, Motion = 180

'Source' 'Sum Sq.' 'd.f.' 'Singular?' 'Mean Sq.' 'F' 'Prob>F' 'Eta2'


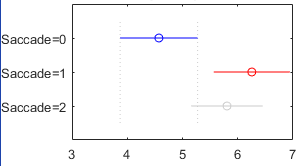
 'Saccade' [ 44.7478] [ 2] [ 0] [ 22.3739] [4.4591] [0.0142] **[0.0902]**

'Error' [451.5820] [ 90] [ 0] [ 5.0176] [] [] [0.9098]

'Total' [496.3298] [ 92] [ 0] [] [] [] []

There is a significant difference reduction in eye speed between Saccades at 0deg and Saccades at 90 deg.

### MONKEY 1: 2-WAY ANOVA Session 1-7, Delay = 300ms

'Source' 'Sum Sq.' 'd.f.' 'Singular?' 'Mean Sq.' 'F' 'Prob>F' 'Eta2'

'Motion' [ 11.2002] [ 1] [ 0] [ 11.2002] [4.8314] [0.0300] [0.0421]

'Saccade' [ 2.5980] [ 2] [ 0] [ 1.2990] [0.5603] [0.5726] [0.0101]

'Motion*Saccade' [ 7.8990] [ 2] [ 0] [ 3.9495] [1.7037] [0.1868] [0.0300]

'Error' [255.0018] [ 110] [ 0] [ 2.3182] [] [] [0.9198]

'Total' [277.2253] [ 115] [ 0] [] [] [] []

There is a significant main effect of motion direction and no interaction, so let’s ignore saccade direction

#### MONKEY 1: 1-WAY ANOVA Session 1-7, Delay = 300ms
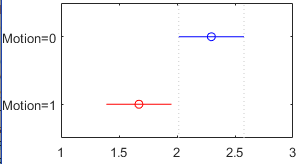
, Ignoring Saccade

'Source' 'Sum Sq.' 'd.f.' 'Singular?' 'Mean Sq.' 'F' 'Prob>F' 'Eta2'

'Motion' [ 11.2962] [ 1] [ 0] [ 11.2962] [4.8425] [0.0298] **[0.0407]**

'Error' [265.9291] [ 114] [ 0] [ 2.3327] [] [] [0.9593]

'Total' [277.2253] [ 115] [ 0] [] [] [] []

There is a significant increase in eye speed when the background moved at 0deg compared to 180deg

## MONKEY 1: 3-WAY ANOVA Session 8-14

'Source' 'Sum Sq.' 'd.f.' 'Singular?' 'Mean Sq.' 'F' 'Prob>F' 'Eta2'

'Delay' [1.4150e+03] [ 1] [ 0] [1.4150e+03] [308.1491] [3.6564e-51] [ 0.4433]

'Motion' [ 123.6566] [ 1] [ 0] [ 123.6566] [ 26.9297] [3.4130e-07] [ 0.0651]

'Saccade' [ 75.6451] [ 2] [ 0] [ 37.8226] [ 8.2369] [3.1390e-04] [ 0.0408]

'Delay*Motion' [ 0.2566] [ 1] [ 0] [ 0.2566] [ 0.0559] [ 0.8133] [1.4438e-04]

'Delay*Saccade' [ 15.9106] [ 2] [ 0] [ 7.9553] [ 1.7325] [ 0.1782] [ 0.0089]

'Motion*Saccade' [ 12.6944] [ 2] [ 0] [ 6.3472] [ 1.3823] [ 0.2522] [ 0.0071]

'Delay*Motion*Sacc…' [ 11.7588] [ 2] [ 0] [ 5.8794] [ 1.2804] [ 0.2791] [ 0.0066]

'Error' [1.7770e+03] [ 387] [ 0] [ 4.5918] [] [] [ 0.5169]

'Total' [3.4381e+03] [ 398] [ 0] [] [] [] []


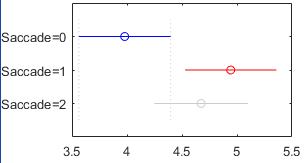
All we have is 3 main effects with no interactions. Therefore the appropriate tests to do are simple effects using 1-way ANOVA of each variable in turn.

#### MONKEY 1: 1-WAY ANOVA examining the main effect of saccade direction.

'Source' 'Sum Sq.' 'd.f.' 'Singular?' 'Mean Sq.' 'F' 'Prob>F' 'Eta2'

'Saccade' [ 66.5692] [ 2] [ 0] [ 33.2846] [3.9094] [0.0208] **[0.0194]**

'Error' [3.3716e+03] [ 396] [ 0] [ 8.5141] [] [] [0.9806]

'Total' [3.4381e+03] [ 398] [ 0] [] [] [] []

There is a significant decrease in the eye speed when the saccade was in the 0deg direction, compared to the 90deg direction.

####
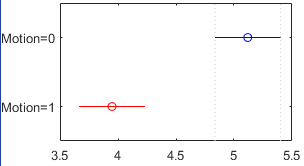
MONKEY 1: 1-WAY ANOVA examining the main effect of motion direction

'Source' 'Sum Sq.' 'd.f.' 'Singular?' 'Mean Sq.' 'F' 'Prob>F' 'Eta2'

'Motion' [ 136.6396] [ 1] [ 0] [136.6396] [16.4307] [6.0735e-05] **[0.0397]**

'Error' [3.3015e+03] [ 397] [ 0] [ 8.3161] [] [] [0.9603]

'Total' [3.4381e+03] [ 398] [ 0] [] [] [] []

There is a significant increase in eye speed when the background moved at 0deg compared to 180deg

#### MONKEY 1: 1-WAY ANOVA examining the main effect of post saccadic delay

'Source' 'Sum Sq.' 'd.f.' 'Singular?' 'Mean Sq.' 'F' 'Prob>F' 'Eta2'

'Delay' [1.4229e+03] [ 1] [ 0] [1.4229e+03] [280.3218] [5.4879e-48] **[0.4139]**

'Error' [2.0152e+03] [ 397] [ 0] [ 5.0761] [] [] [0.5861]


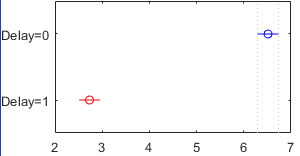
 'Total' [3.4381e+03] [ 398] [ 0] [] [] [] []

There is a significant increase in eye speed when post-saccadic delay was 50ms compared to 300ms

## MONKEY 1: 3-WAY ANOVA Session 15-21

'Source' 'Sum Sq.' 'd.f.' 'Singular?' 'Mean Sq.' 'F' 'Prob>F' 'Eta2'

'Delay' [1.6577e+03] [ 1] [ 0] [1.6577e+03] [238.4789] [7.2678e-45] [0.3052]

'Motion' [ 805.0581] [ 1] [ 0] [ 805.0581] [115.8139] [1.2949e-24] [0.1758]

'Saccade' [ 32.6646] [ 2] [ 0] [ 16.3323] [ 2.3495] [ 0.0964] [0.0086]

'Delay*Motion' [ 6.3136] [ 1] [ 0] [ 6.3136] [ 0.9083] [ 0.3410] [0.0017]

'Delay*Saccade' [ 36.9316] [ 2] [ 0] [ 18.4658] [ 2.6564] [ 0.0711] [0.0097]

'Motion*Saccade' [ 78.0850] [ 2] [ 0] [ 39.0425] [ 5.6166] [ 0.0039] [0.0203]

'Delay*Motion*Sacc…' [ 58.7084] [ 2] [ 0] [ 29.3542] [ 4.2228] [ 0.0151] [0.0153]

'Error' [3.7746e+03] [ 543] [ 0] [ 6.9513] [] [] [0.5830]

'Total' [6.4738e+03] [ 554] [ 0] [] [] [] []

Since there is no interaction due to delay let’s have a look at the data at each delay

### MONKEY 1: 2-WAY ANOVA Session 15-21, Delay = 50ms

'Source' 'Sum Sq.' 'd.f.' 'Singular?' 'Mean Sq.' 'F' 'Prob>F' 'Eta2'

'Motion' [ 273.3622] [ 1] [ 0] [273.3622] [30.5722] [9.5843e-08] [0.1281]

'Saccade' [ 56.6870] [ 2] [ 0] [ 28.3435] [ 3.1699] [ 0.0440] [0.0296]

'Motion*Saccade' [ 106.9594] [ 2] [ 0] [ 53.4797] [ 5.9811] [ 0.0030] [0.0544]


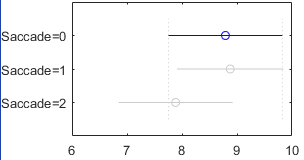
 'Error' [1.8598e+03] [ 208] [ 0] [ 8.9415] [] [] [0.8091]

'Total' [2.2988e+03] [ 213] [ 0] [] [] [] []

Since there is an interaction between Motion & Saccade let’s have a look at the data for each motion direction

#### MONKEY 1: 1-WAY ANOVA Session 15-21, Delay = 50ms, Motion = 0

'Saccade' [ 21.1659] [ 2] [ 0] [ 10.5830] [0.8203] [0.4431] **[0.0155]**

'Error' [1.3417e+03] [ 104] [ 0] [ 12.9011] [] [] [0.984 5]

'Total' [1.3629e+03] [ 106] [ 0] [] [] [] []

There is no effect of saccade direction when the motion of the background is at 0deg

####
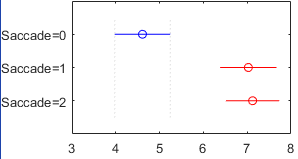
MONKEY 1: 1-WAY ANOVA Session 15-21, Delay = 50ms, Motion = 180

'Saccade' [143.6152] [ 2] [ 0] [ 71.8076] [14.4137] [2.9838e-06] **[0.2170]**

'Error' [518.1170] [ 104] [ 0] [ 4.9819] [] [] [0.7830]

'Total' [661.7322] [ 106] [ 0] [] [] [] []

The is a significant decrease in eye speed when the saccade was at 0deg when compared to 90 or 180deg.

### MONKEY 1: 2-WAY ANOVA Session 15-21, Delay = 300ms

'Source' 'Sum Sq.' 'd.f.' 'Singular?' 'Mean Sq.' 'F' 'Prob>F' 'Eta2'

'Motion' [ 614.0756] [ 1] [ 0] [614.0756] [107.4387] [5.0948e-22] [ 0.2428]

'Saccade' [ 0.8392] [ 2] [ 0] [ 0.4196] [ 0.0734] [ 0.9292] [4.3811e-04]

'Motion*Saccade' [ 2.4245] [ 2] [ 0] [ 1.2122] [ 0.2121] [ 0.8090] [ 0.0013]

'Error' [1.9147e+03] [ 335] [ 0] [ 5.7156] [] [] [ 0.7515]

'Total' [2.5478e+03] [ 340] [ 0] [] [] [] []

There is a significant main effect of motion direction so let’s ignore saccade direction

####
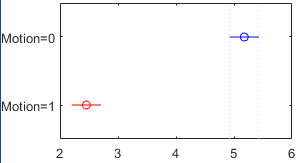
MONKEY 1: 1-WAY ANOVA Session 1-7, Delay = 300ms, Ignoring Saccade

'Source' 'Sum Sq.' 'd.f.' 'Singular?' 'Mean Sq.' 'F' 'Prob>F' 'Eta2'

'Motion' [ 629.8389] [ 1] [ 0] [629.8389] [111.3220] [1.0786e-22] **[0.2472]**

'Error' [1.9180e+03] [ 339] [ 0] [ 5.6578] [] [] [0.7528]

'Total' [2.5478e+03] [ 340] [ 0] [] [] [] []

There is a significant increase in eye speed when the background moved at 0deg compared to 180deg

# MONKEY 2: 4-WAY ANOVA

'Source' 'Sum Sq.' 'd.f.' 'Singular?' 'Mean Sq.' 'F' 'Prob>F' 'Eta2' 'Partial Eta2'

'Delay' [5.3906e+03] [ 1] [ 0] [5.3906e+03] [2.8385e+03] [ 0] [ 0.2925] [ 0.4614]

'Motion' [4.9312e+03] [ 1] [ 0] [4.9312e+03] [2.5966e+03] [ 0] [ 0.2675] [ 0.4394]

'Saccade' [ 130.7157] [ 2] [ 0] [ 65.3578] [ 34.4148] [1.6106e-15] [ 0.0071] [ 0.0204]

'Time' [ 520.7512] [ 2] [ 0] [ 260.3756] [ 137.1033] [6.2067e-58] [ 0.0283] [ 0.0764]

'Delay*Motion' [ 332.0184] [ 1] [ 0] [ 332.0184] [ 174.8275] [6.2199e-39] [ 0.0180] [ 0.0501]

'Delay*Saccade' [ 36.3968] [ 2] [ 0] [ 18.1984] [ 9.5825] [7.0851e-05] [ 0.0020] [ 0.0058]

'Delay*Time' [ 0.9568] [ 2] [ 0] [ 0.4784] [ 0.2519] [ 0.7773] [5.1913e-05] [ 1.5205e-04]

'Motion*Saccade' [ 385.9568] [ 2] [ 0] [ 192.9784] [ 101.6146] [1.4790e-43] [ 0.0209] [ 0.0578]

'Motion*Time' [ 394.2643] [ 2] [ 0] [ 197.1321] [ 103.8018] [1.8859e-44] [ 0.0214] [ 0.0590]

'Saccade*Time' [ 19.3835] [ 4] [ 0] [ 4.8459] [ 2.5516] [ 0.0373] [ 0.0011] [ 0.0031]

'Delay*Motion*Sacc…' [ 107.7259] [ 2] [ 0] [ 53.8630] [ 28.3621] [6.1203e-13] [ 0.0058] [ 0.0168]

'Delay*Motion*Time' [ 8.0392] [ 2] [ 0] [ 4.0196] [ 2.1166] [ 0.1206] [4.3617e-04] [ 0.0013]

'Delay*Saccade*Time' [ 13.7339] [ 4] [ 0] [ 3.4335] [ 1.8079] [ 0.1244] [7.4515e-04] [ 0.0022]

'Motion*Saccade*Time' [ 13.1812] [ 4] [ 0] [ 3.2953] [ 1.7352] [ 0.1393] [7.1516e-04] [ 0.0021]

'Delay*Motion*Sacc…' [ 6.8184] [ 4] [ 0] [ 1.7046] [ 0.8976] [ 0.4644] [3.6994e-04] [ 0.0011]

'Error' [6.2918e+03] [3313] [ 0] [ 1.8991] [] [] [ 0.3414] []

'Total' [1.8431e+04] [3348] [ 0] [] [] [] [] []

As we have a 3-way interaction that doesn’t involve Time(Sessions) and to allow comparison with the results of Monkey 1: let’s examine what the data looks like at each time(Sessions).

## MONKEY 2: 3-WAY ANOVA Session 1-7

'Source' 'Sum Sq.' 'd.f.' 'Singular?' 'Mean Sq.' 'F' 'Prob>F' 'Eta2'

'Delay' [2.3994e+03] [ 1] [ 0] [2.3994e+03] [1.5901e+03] [2.9639e-232] [0.5343]

'Motion' [ 844.6067] [ 1] [ 0] [ 844.6067] [ 559.7247] [3.2305e-104] [0.2877]

'Saccade' [ 33.4144] [ 2] [ 0] [ 16.7072] [ 11.0719] [ 1.6964e-05] [0.0157]

'Delay*Motion' [ 124.0719] [ 1] [ 0] [ 124.0719] [ 82.2230] [ 4.0439e-19] [0.0560]

'Delay*Saccade' [ 10.6239] [ 2] [ 0] [ 5.3119] [ 3.5202] [ 0.0299] [0.0051]

'Motion*Saccade' [ 166.8167] [ 2] [ 0] [ 83.4083] [ 55.2751] [ 8.0105e-24] [0.0739]

'Delay*Motion*Sacc…' [ 35.5329] [ 2] [ 0] [ 17.7665] [ 11.7739] [ 8.5036e-06] [0.0167]

'Error' [2.0914e+03] [1386] [ 0] [ 1.5090] [] [] [0.3701]

'Total' [5.6515e+03] [1397] [ 0] [] [] [] []

Since everything is significant lets replicate the analysis from MONKEY 1: looking at each delay separately

### MONKEY 2: 2-WAY ANOVA Session 1-7, Delay = 50ms

'Source' 'Sum Sq.' 'd.f.' 'Singular?' 'Mean Sq.' 'F' 'Prob>F' 'Eta2'


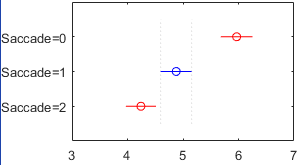
 'Motion' [ 802.4126] [ 1] [ 0] [802.4126] [353.1753] [9.6553e-64] [0.3425]

'Saccade' [ 37.6993] [ 2] [ 0] [ 18.8496] [ 8.2965] [2.7558e-04] [0.0239]

'Motion*Saccade' [ 166.7959] [ 2] [ 0] [ 83.3979] [ 36.7069] [7.3084e-16] [0.0977]

'Error' [1.5404e+03] [ 678] [ 0] [ 2.2720] [] [] [0.6115]

'Total' [2.5189e+03] [ 683] [ 0] [] [] [] []

Since there is an interaction between Motion & Saccade let’s have a look at the data for each motion direction

#### MONKEY 2: 1-WAY ANOVA Session 1-7m Delay = 50ms, Motion = 0

'Source' 'Sum Sq.' 'd.f.' 'Singular?' 'Mean Sq.' 'F' 'Prob>F' 'Eta2'

'Saccade' [ 170.1541] [ 2] [ 0] [ 85.0770] [26.7155] [1.7176e-11] **[0.1376]**

'Error' [1.0668e+03] [ 335] [ 0] [ 3.1846] [] [] [0.8624]


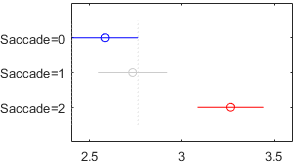
 'Total' [1.2370e+03] [ 337] [ 0] [] [] [] []

There is a significant reduction in eye speed between Saccades at 180deg and 90deg, and a significant

increase in eye speed between saccades at 0 and 90degs.

#### MONKEY 2: 1-WAY ANOVA Session 1-7m Delay = 50ms, Motion = 180

'Source' 'Sum Sq.' 'd.f.' 'Singular?' 'Mean Sq.' 'F' 'Prob>F' 'Eta2'

'Saccade' [ 29.9703] [ 2] [ 0] [ 14.9852] [10.8531] [2.6895e-05] **[0.0595]**

'Error' [473.5876] [ 343] [ 0] [ 1.3807] [] [] [0.9405]

'Total' [503.5580] [ 345] [ 0] [] [] [] []

There is a significant increase in eye speed between saccades at 180deg and those at 0 and 90deg.

###
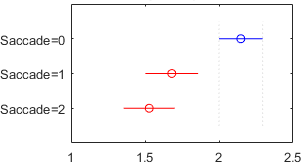
MONKEY 2: 2-WAY ANOVA Session 1-7, Delay = 300ms

'Source' 'Sum Sq.' 'd.f.' 'Singular?' 'Mean Sq.' 'F' 'Prob>F' 'Eta2'

'Motion' [161.7609] [ 1] [ 0] [161.7609] [207.8462] [1.6724e-41] [0.2269]

'Saccade' [ 3.9142] [ 2] [ 0] [ 1.9571] [ 2.5147] [ 0.0816] [0.0071]

'Motion*Saccade' [ 29.7528] [ 2] [ 0] [ 14.8764] [ 19.1146] [8.2221e-09] [0.0512]

'Error' [551.0169] [ 708] [ 0] [ 0.7783] [] [] [0.7103]

'Total' [775.7396] [ 713] [ 0] [] [] [] []

Since there is an interaction between Motion & Saccade let’s have a look at the data for each motion direction

#### MONKEY 2: 1-WAY ANOVA Session 1-7m Delay = 300ms, Motion = 0

'Source' 'Sum Sq.' 'd.f.' 'Singular?' 'Mean Sq.' 'F' 'Prob>F' 'Eta2'

'Saccade' [ 27.4010] [ 2] [ 0] [ 13.7005] [11.6418] [1.2625e-05] **[0.0609]**


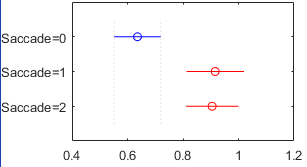
 'Error' [422.4842] [ 359] [ 0] [ 1.1768] [] [] [0.9391]

'Total' [449.8852] [ 361] [ 0] [] [] [] []

There is a significant increase in eye speed between saccades at 0deg and those at 90 and 180deg.

#### MONKEY 2: 1-WAY ANOVA Session 1-7m Delay = 300ms, Motion = 180

'Source' 'Sum Sq.' 'd.f.' 'Singular?' 'Mean Sq.' 'F' 'Prob>F' 'Eta2'

'Saccade' [ 6.4174] [ 2] [ 0] [ 3.2087] [8.7124] [2.0308e-04] **[0.0476]**

'Error' [128.5327] [ 349] [ 0] [ 0.3683] [] [] [0.9524]

'Total' [134.9501] [ 351] [ 0] [] [] [] []

There is a significant decrease in eye speed between saccades at 0deg and those at 0 and 180deg.

## MONKEY 2: 3-WAY ANOVA Session 8-14

'Source' 'Sum Sq.' 'd.f.' 'Singular?' 'Mean Sq.' 'F' 'Prob>F' 'Eta2'

'Delay' [1.8111e+03] [ 1] [ 0] [1.8111e+03] [773.4976] [2.3299e-128] [0.4212]

'Motion' [2.0326e+03] [ 1] [ 0] [2.0326e+03] [868.1123] [5.7203e-140] [0.4495]

'Saccade' [ 49.9753] [ 2] [ 0] [ 24.9877] [ 10.6720] [ 2.5771e-05] [0.0197]

'Delay*Motion' [ 78.2045] [ 1] [ 0] [ 78.2045] [ 33.4003] [ 9.8486e-09] [0.0305]

'Delay*Saccade' [ 4.8143] [ 2] [ 0] [ 2.4071] [ 1.0281] [ 0.3581] [0.0019]

'Motion*Saccade' [ 104.5838] [ 2] [ 0] [ 52.2919] [ 22.3333] [ 3.1550e-10] [0.0403]

'Delay*Motion*Sacc…' [ 20.3463] [ 2] [ 0] [ 10.1731] [ 4.3448] [ 0.0132] [0.0081]

'Error' [2.4889e+03] [1063] [ 0] [ 2.3414] [] [] [0.3848]

'Total' [6.4681e+03] [1074] [ 0] [] [] [] []

Lets replicate the analysis from the first 7 sessions as nearly everything is significant. We will look at each delay separately

### MONKEY 2: 2-WAY ANOVA Session 8-14, Delay = 50ms

'Source' 'Sum Sq.' 'd.f.' 'Singular?' 'Mean Sq.' 'F' 'Prob>F' 'Eta2'

'Motion' [1.3160e+03] [ 1] [ 0] [1.3160e+03] [414.0398] [8.5945e-67] [0.4636]


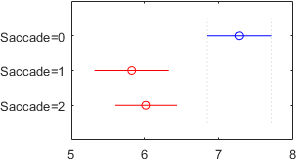
 'Saccade' [ 36.5332] [ 2] [ 0] [ 18.2666] [ 5.7470] [ 0.0034] [0.0234]

'Motion*Saccade' [ 87.5701] [ 2] [ 0] [ 43.7851] [ 13.7756] [1.5243e-06] [0.0544]

'Error' [1.5225e+03] [ 479] [ 0] [ 3.1785] [] [] [0.5108]

'Total' [2.9803e+03] [ 484] [ 0] [] [] [] []

Since there is an interaction between Motion & Saccade let’s have a look at the data for each motion direction

#### MONKEY 2: 1-WAY ANOVA Session 8-14, Delay = 50ms, Motion = 0

'Source' 'Sum Sq.' 'd.f.' 'Singular?' 'Mean Sq.' 'F' 'Prob>F' 'Eta2'

'Saccade' [ 92.4174] [ 2] [ 0] [ 46.2087] [8.5472] [2.6616e-04] **[0.0721]**

'Error' [1.1894e+03] [ 220] [ 0] [ 5.4063] [] [] [0.9279]

'Total' [1.2818e+03] [ 222] [ 0] [] [] [] []


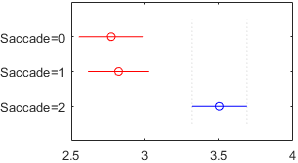
There is a significant increase in eye speed between saccades at 0deg and those at 90 and 180deg

#### MONKEY 2: 1-WAY ANOVA Session 8-14, Delay = 50ms, Motion = 180

'Source' 'Sum Sq.' 'd.f.' 'Singular?' 'Mean Sq.' 'F' 'Prob>F' 'Eta2'

'Saccade' [ 31.2506] [ 2] [ 0] [ 15.6253] [12.1495] [9.0491e-06] **[0.0858]**

'Error' [333.0971] [ 259] [ 0] [ 1.2861] [] [] [0.9142]

'Total' [364.3476] [ 261] [ 0] [] [] [] []

There is a significant increase in eye speed between saccades at 180deg and those at 90 and 0deg

### MONKEY 2: 2-WAY ANOVA Session 8-14, Delay = 300ms

'Source' 'Sum Sq.' 'd.f.' 'Singular?' 'Mean Sq.' 'F' 'Prob>F' 'Eta2'


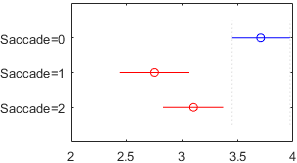
 'Motion' [ 733.7120] [ 1] [ 0] [733.7120] [443.3596] [1.1740e-73] [0.4316]

'Saccade' [ 15.8610] [ 2] [ 0] [ 7.9305] [ 4.7922] [ 0.0086] [0.0161]

'Motion*Saccade' [ 30.9932] [ 2] [ 0] [ 15.4966] [ 9.3641] [9.9325e-05] [0.0311]

'Error' [ 966.4567] [ 584] [ 0] [ 1.6549] [] [] [0.5399]

'Total' [1.7899e+03] [ 589] [ 0] [] [] [] []

Since there is an interaction between Motion & Saccade let’s have a look at the data for each motion direction

#### MONKEY 2: 1-WAY ANOVA Session 8-14, Delay = 300ms, Motion = 0

'Source' 'Sum Sq.' 'd.f.' 'Singular?' 'Mean Sq.' 'F' 'Prob>F' 'Eta2'

'Saccade' [ 45.3750] [ 2] [ 0] [ 22.6875] [8.1999] [3.4371e-04] **[0.0537]**

'Error' [799.6025] [ 289] [ 0] [ 2.7668] [] [] [0.9463]

'Total' [844.9775] [ 291] [ 0] [] [] [] []

There is a significant increase in eye speed between saccades at 0deg and those at 90 and 180deg

####
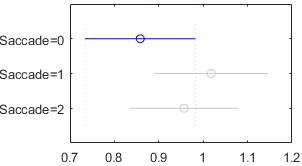
MONKEY 2: 1-WAY ANOVA Session 8-14, Delay = 300ms, Motion = 180

'Source' 'Sum Sq.' 'd.f.' 'Singular?' 'Mean Sq.' 'F' 'Prob>F' 'Eta2'

'Saccade' [ 1.2680] [ 2] [ 0] [ 0.6340] [1.1209] [0.3274] **[0.0075]**

'Error' [166.8542] [ 295] [ 0] [ 0.5656] [] [] [0.9925]

'Total' [168.1221] [ 297] [ 0] [] [] [] []

There is no significant difference in eye speed due to saccade direction

## MONKEY 2: 3-WAY ANOVA Session 15-21

'Source' 'Sum Sq.' 'd.f.' 'Singular?' 'Mean Sq.' 'F' 'Prob>F' 'Eta2'

'Delay' [1.4094e+03] [ 1] [ 0] [1.4094e+03] [ 711.5289] [7.7859e-115] [0.4516]

'Motion' [2.1155e+03] [ 1] [ 0] [2.1155e+03] [1.0680e+03] [3.8156e-153] [0.5528]

'Saccade' [ 58.0094] [ 2] [ 0] [ 29.0047] [ 14.6429] [ 5.5730e-07] [0.0328]

'Delay*Motion' [ 136.4429] [ 1] [ 0] [ 136.4429] [ 68.8825] [ 3.9998e-16] [0.0738]

'Delay*Saccade' [ 31.8151] [ 2] [ 0] [ 15.9076] [ 8.0308] [ 3.5016e-04] [0.0183]

'Motion*Saccade' [ 134.2625] [ 2] [ 0] [ 67.1312] [ 33.8908] [ 6.7642e-15] [0.0727]

'Delay*Motion*Sacc…' [ 55.4052] [ 2] [ 0] [ 27.7026] [ 13.9855] [ 1.0530e-06] [0.0314]

'Error' [1.7114e+03] [ 864] [ 0] [ 1.9808] [] [] [0.2966]

'Total' [5.7704e+03] [ 875] [ 0] [] [] [] []

Since everything is significant lets replicate the analysis from MONKEY 1: looking at each delay separately

### MONKEY 2: 2-WAY ANOVA Session 15-21, Delay = 50ms

'Source' 'Sum Sq.' 'd.f.' 'Singular?' 'Mean Sq.' 'F' 'Prob>F' 'Eta2'

'Motion' [1.7957e+03] [ 1] [ 0] [1.7957e+03] [717.0176] [4.1334e-96] [0.6076]

'Saccade' [ 93.1083] [ 2] [ 0] [ 46.5541] [ 18.5891] [1.7164e-08] [0.0743]

'Motion*Saccade' [ 189.5133] [ 2] [ 0] [ 94.7566] [ 37.8364] [6.0290e-16] [0.1405]


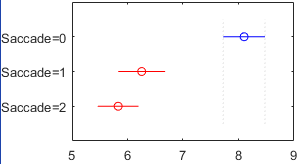
 'Error' [1.1595e+03] [ 463] [ 0] [ 2.5044] [] [] [0.3550]

'Total' [3.2665e+03] [ 468] [ 0] [] [] [] []

Since there is an interaction between Motion & Saccade let’s have a look at the data for each motion direction

#### MONKEY 2: 1-WAY ANOVA Session 15-21, Delay = 50ms, Motion = 0

'Source' 'Sum Sq.' 'd.f.' 'Singular?' 'Mean Sq.' 'F' 'Prob>F' 'Eta2'

'Saccade' [ 232.5560] [ 2] [ 0] [116.2780] [28.1718] [1.2061e-11] **[0.2003]**

'Error' [ 928.6798] [ 225] [ 0] [ 4.1275] [] [] [0.7997]

'Total' [1.1612e+03] [ 227] [ 0] [] [] [] []

There is a significant increase in eye speed between saccades at 0deg and those at 90 and 180deg

####
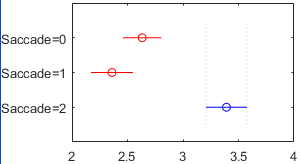
MONKEY 2: 1-WAY ANOVA Session 15-21, Delay = 50ms, Motion = 180

'Source' 'Sum Sq.' 'd.f.' 'Singular?' 'Mean Sq.' 'F' 'Prob>F' 'Eta2'

'Saccade' [ 44.1157] [ 2] [ 0] [ 22.0579] [22.7414] [9.1619e-10] **[0.1604]**

'Error' [230.8467] [ 238] [ 0] [ 0.9699] [] [] [0.8396]

'Total' [274.9624] [ 240] [ 0] [] [] [] []

There is a significant increase in eye speed between saccades at 0deg and those at 90 and 180deg

### MONKEY 2: 2-WAY ANOVA Session 15-21, Delay = 300ms

'Source' 'Sum Sq.' 'd.f.' 'Singular?' 'Mean Sq.' 'F' 'Prob>F' 'Eta2'

'Motion' [ 548.2651] [ 1] [ 0] [548.2651] [398.3651] [4.7895e-62] [0.4984]


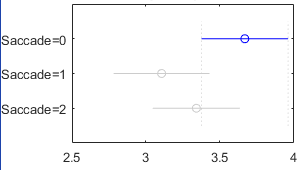
 'Saccade' [ 3.9320] [ 2] [ 0] [ 1.9660] [ 1.4285] [ 0.2409] [0.0071]

'Motion*Saccade' [ 10.5797] [ 2] [ 0] [ 5.2898] [ 3.8435] [ 0.0222] [0.0188]

'Error' [ 551.8915] [ 401] [ 0] [ 1.3763] [] [] [0.4887]

'Total' [1.1292e+03] [ 406] [ 0] [] [] [] []

Since there is an interaction between Motion & Saccade let’s have a look at the data for each motion direction

#### MONKEY 2: 1-WAY ANOVA Session 15-21, Delay = 300ms, Motion = 0

'Source' 'Sum Sq.' 'd.f.' 'Singular?' 'Mean Sq.' 'F' 'Prob>F' 'Eta2'

'Saccade' [ 10.5141] [ 2] [ 0] [ 5.2570] [2.3432] [0.0987] **[0.0230]**

'Error' [446.4670] [ 199] [ 0] [ 2.2436] [] [] [0.9770]

'Total' [456.9811] [ 201] [ 0] [] [] [] []

There is no significant difference in eye speed due to saccade direction

####
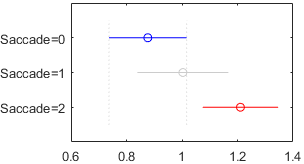
MONKEY 2: 1-WAY ANOVA Session 15-21, Delay = 300ms, Motion = 180

'Source' 'Sum Sq.' 'd.f.' 'Singular?' 'Mean Sq.' 'F' 'Prob>F' 'Eta2'

'Saccade' [ 4.2851] [ 2] [ 0] [ 2.1426] [4.1053] [0.0179] **[0.0391]**

'Error' [105.4246] [ 202] [ 0] [ 0.5219] [] [] [0.9609]

'Total' [109.7097] [ 204] [ 0] [] [] [] []

There is a significant difference in eye speeds between Saccades at 0deg and 180deg.
